# Multimodal monitoring: practical recommendations (dos and don'ts) in challenging situations and uncertainty

**DOI:** 10.3389/fneur.2023.1135406

**Published:** 2023-05-03

**Authors:** Rohan Sharma, Mariam Tsikvadze, Jeffrey Peel, Levi Howard, Nidhi Kapoor, William D. Freeman

**Affiliations:** ^1^Department of Neurology, Mayo Clinic in Florida, Jacksonville, FL, United States; ^2^Department of Neurology, Baptist Medical Center, Jacksonville, FL, United States

**Keywords:** multimodal monitoring, pupillometry, transcranial Doppler, Near Infrared Spectroscopy, quantitative EEG, cerebral blood flow monitoring, intracranial pressure monitoring, cerebral metabolism monitoring

## Abstract

With the advancements in modern medicine, new methods are being developed to monitor patients in the intensive care unit. Different modalities evaluate different aspects of the patient's physiology and clinical status. The complexity of these modalities often restricts their use to the realm of clinical research, thereby limiting their use in the real world. Understanding their salient features and their limitations can aid physicians in interpreting the concomitant information provided by multiple modalities to make informed decisions that may affect clinical care and outcomes. Here, we present a review of the commonly used methods in the neurological intensive care unit with practical recommendations for their use.

## 1. Introduction

Multimodal monitoring (MMM) is often used in patients with severe brain injury when there is no reliable neurological examination to follow or when there is a specific question to be answered leading to a particular intervention. MMM in brain injury has revealed important physiological information such as intracranial autoregulation relationships and optimal cerebral perfusion pressure. MMM involves the use of multiple modalities to monitor neurophysiology, often with multiple probes or sensors. There are different types of MMM methods, which are topically reviewed here, with challenging situations and practical recommendations for certain types of neurological patient population.

## 2. Modalities

### 2.1. Physical examination

A detailed neurological examination is of paramount importance in the evaluation and monitoring of patients in the neurological intensive care unit. Subtle changes in the neurological examination can indicate a potential clinical disaster and guide physicians to targeted neuroimaging and/or appropriate and timely neuro-intervention to prevent secondary brain injury. However, the modalities of the neurological examination in the ICU are markedly different from those of the general neurological examination in the outpatient setting. Often in the ICU, the examination is limited to the assessment of the level of consciousness, a limited language examination because the patient is intubated, and the observation of cranial nerve function if the patient is unable to follow complex commands. The motor function may be limited to noxious stimulation or observation of motor responses and comparison of reflexes for symmetry or changes over time due to an underlying or evolving brain or spinal cord injury. It is therefore imperative that serial and detailed neurological examinations be consistently performed and documented to avoid confusion and unnecessary testing. Standardized examinations are also useful, such as the NIH Stroke Scale, Glasgow Coma Scale (GCS), or our FOUR Scale, which have good inter-rater reliability. Changes in examinations are also often confounded by the milieu of medications used in the ICU, which should be considered when examining patients. A daily sedation holiday, such as the ABCDE bundle, should be considered in all neuro-ICU patients unless intracranial pressure (ICP) is unstable or there is a good reason not to temporarily withhold medication. The deficits on examination may be global or diffuse within the central nervous system (e.g., coma or encephalopathy, etc.), localized (e.g., hemiparesis, etc.), or generalized peripheral findings (e.g., myopathy, etc.). An experienced examiner can use the deficits to localize the examination within the central and/or peripheral nervous systems, thereby making decisions about appropriate investigations and subsequent management. GCS, motor examination, and pupillary examination changes have been shown to be strong prognostic indicators for outcomes in patients with traumatic brain injury (TBI) (1, 2). However, studies have also indicated that brainstem reflexes in conjunction with motor response add prognostic value to the physical examination of brain injury (3, 4).

Clinical interpretation of common neurological deficits:

#### 2.1.1. Impaired level of consciousness

Consciousness is impaired when a bilateral ascending reticular formation is involved. Because the reticular formation is widely distributed in both hemispheres, consciousness is affected by large territorial lesions of the cortex. Hemispheric lesions may cause drowsiness, but severe impairment such as stupor or coma occurs with disease processes involving bilateral cortical hemispheres, bilateral thalami, bilateral midbrain, and pons. Consciousness may also be impaired by systemic processes such as metabolic derangements (hyponatremia, uremia, hyperammonemia, etc.). However, lesions of the central nervous system are often accompanied by other deficits on examination.

#### 2.1.2. Decreased responsiveness

Decreased responsiveness may occur either with impaired consciousness (as discussed above) or with preserved consciousness. Acutely decreased responsiveness in awake patients requires further assessment of the underlying deficit. It may be a manifestation of global aphasia, bradyphrenia, abulia, catatonia, unilateral or bilateral hemineglect, or locked-in syndrome (see Table 1 for localization and clinical interpretation).

**Table 1:** Clinical examination, interpretation, and clinic correlates.

**Symptom**	**Clinical examination**	**Interpretation**	**Clinical correlate**
Impaired consciousness	Somnolence, lethargy, stupor, coma	Impairment of the bilateral reticular activating system, large bihemispheric lesions, bithalamic lesions, and bilateral brain stem lesions	Bilateral strokes/hemorrhage, hydrocephalus, status epilepticus, severe metabolic derangements
Decreased responsiveness with preserved consciousness	Aphasia^*^ Abulia/akinetic mutism^+^ Unilateral or bilateral hemineglects Locked-in^ψ^ Syndrome Catatonia^ϵ^	^*^Dominant frontal (MCA territory) lesion ^+^Medial frontal (ACA) lesion Parietal lobe lesions, ^ψ^Central Pontine lesion ^ϵ^Psychomotor slowing/avolition	^*^+ ψ Strokes, space-occupying lesions (SOL) e.g., hemorrhage/tumors/infection in the CNS ^ϵ^Psychiatric diseases, autoimmune/infectious encephalitis
Gaze deviation	Lateral^∞^ Vertical^μ^	^∞^Frontal eye fields, Parietal eye fields, paramedian pontine reticular formation, vestibular nuclei, vermis flocculonodular lobes ^μ^Dorsal midbrain and medial thalami	^∞^Unilateral SOL, status epilepticus ^μ^SOL in dorsal midbrain, hydrocephalus, oculogyric crisis
Pupillary changes	Small circular^α^ Large circular^β^ Oval	^α^Horner syndrome (damage to the sympathetic chain: hypothalamic, pontine, lateral medullary, cervical spinal lesions, and carotid injury) ^β^Oculomotor nerve or midbrain compression Increased ICP	^α^TBI, SAH ^β^Uncal herniation, posterior communicating artery aneurysm, vitreal/retinal hemorrhage, retinal detachment, optic neuropathy ^α^βDrug effect Transtentorial herniation
Posturing	Flexor Extensor	Midbrain Lesion Pontine and medullary lesions	Stroke, SOL, encephalitis
Muscular tone changes	Spasticity^£^ Flaccidity^*e*^	^£^CNS (cortex, subcortex, brain stem, spinal) lesions ^e^Spinal cord, nerve root, peripheral nerves, neuromuscular junction	^£^Stroke, SOL ^e^Stroke, SOL, AIDP, MG

#### 2.1.3. Gaze deviation

Acute forced conjugate gaze deviation has localizing value and may be seen in hemispheric lesions involving the frontal eye field and manifesting contralateral to the lesion, but may also occur with lesions of the basal ganglia, parietal eye fields, neighboring temporoparietal cortical regions, pontine lesions involving the paramedian pontine reticular formation; lateral medulla by involving the vestibular nuclei; and vermis and flocculonodular lobes of the cerebellum (5–7). Vertical gaze deviation, often referred to as Parinaud's syndrome, is localized to the dorsal midbrain and medial thalami (8, 9). In the neuro-ICU, acute vertical gaze deviation should raise the alarm for pressure on the rostral midbrain, as seen in acute hydrocephalus or midbrain hemorrhage. Rarely, acute vertical gaze deviation may be seen in oculogyric crisis secondary to drugs acting on dopamine receptors (10).

#### 2.1.4. Pupillary changes

The pupillary reflex is mediated by the optic nerve (afferent) and the oculomotor nerve (efferent). The pretectal and Edinger-Westphal nuclei are brainstem relays for the reflex, while the ciliary (parasympathetic) and superior cervical (sympathetic) ganglia are peripheral relays. Sudden changes in pupil size are often a cause for concern in ICU patients. Both miosis and mydriasis are clinically important, but pupillary symmetry and reactivity have better-localizing values (Table 1).

#### 2.1.5. Reflexes

In ICU patients with impaired consciousness, general cranial nerve examination, motor examination, and sensory examination are often limited, and assessment of neurologic function is limited to examination of reflexes. The pupillary or light reflex is discussed in detail above. The blink reflex is a cortical reflex mediated by the optic nerve (afferent) and facial nerve (efferent), in which a visual stimulus, such as the waving of a hand, elicits an eye blink. Intact visual pathways and the cortex must be intact for this reflex to be present. Similarly, the acoustic reflex elicits an eye blink with a sudden loud sound. It is mediated by the vestibulocochlear nerve (afferent) and the facial nerve (efferent), but the reflex arc involves only the brainstem (11). Other brainstem reflexes are the corneal reflex, mediated by the trigeminal nerve (afferent) and the facial nerve (efferent); the gag reflex, mediated by the glossopharyngeal nerve (afferent) and the vagus nerve (efferent); and the cough reflex, mediated by the vagus nerve (afferent and efferent). The vestibulo-ocular and oculo-cephalic reflexes are complex multisegmental reflexes mediated by the vestibulocochlear nerve (afferent) and the oculomotor, abducens, and trochlear nerves (efferent), with the interplay of multiple nuclei and internuclear tracts. The corneal reflex is assessed by gently touching the cornea (either with a cotton swab or water) which elicits a blink. The gag reflex is elicited by touching the back of the pharynx with a spatula, which should produce visible palatal elevation and gag response. The cough reflex is elicited by suctioning the trachea of a mechanically ventilated patient, which should produce a visible cough response. The oculo-cephalic reflex can be elicited by gently rotating the head horizontally and vertically, causing the gaze to be directed to the opposite side (which can be suppressed in an awake patient). The vestibulo-ocular or caloric reflex can be elicited by elevating the patient's head to 30 degrees and injecting ice-cold water into the external auditory canal. A preserved reflex would manifest as nystagmus with a fast component to the opposite ear and a slow component to the same ear. Loss of these reflexes may localize the involvement of parts of the brain/brainstem that contain the nuclear centers involved in these reflexes. The degree of sedation and/or use of paralytic agents should also be considered in the evaluation, as they may also suppress these reflexes.

#### 2.1.6. Motor examination

Motor examination in the ICU is often reflexive because patients are unable to follow commands. Localization or withdrawal in response to pain is a relatively good prognostic sign. Flexor and extensor posturing are poor prognostic indicators and suggest a lesion above or below the midbrain, respectively. These four signs are also part of the motor domain of the Glasgow Coma Scale. Involuntary motor activity may indicate an underlying metabolic disorder (asterixis, myoclonus, tremor, etc.) or seizures (stereotyped rhythmic movements, generalized myoclonus, non-reflexive tonic contractions, etc.). Loss of muscle tone, including those of the bowel and bladder, suggests an acute spinal cord pathology. Neuromuscular disorders such as myasthenia gravis (MG), AIDP (acute inflammatory demyelinating polyneuropathy), ALS (amyotrophic lateral sclerosis), etc. may present with bulbar weakness, often manifesting as dysphagia and difficulty with extubation. Deep tendon reflexes may be decreased in AIDP, neuromuscular blockade, botulism, magnesium toxicity, or spinal shock.

##### 2.1.6.1. Dos

A detailed neurologic examination should be performed and documented at the time of the patient's initial assessment, and any changes in the examination should also be documented. This is imperative to ensure that any changes in the examination are new and warrant further evaluation. Healthcare providers should have a differential diagnosis and intervention plan before ordering any further tests, including imaging. Medications often confound the interpretation of tests in the ICU and should always be considered with any examination (Table 2).

**Table 2:** Summary of modality use and limitations.

**Modality**	**Use**	**Advantages**	**Limitations**
Physical examination	All neurological disorders	Localization and targeting merit further investigation Cost-effective Can pick up early signs of neurological decline	Can interfere with sleep hygiene Affected by medications used in the ICU
Pupillometry	All neurological disorders	Objective, limited inter-examiner variability	Affected by ambient light and limited by local factors (irregular pupils, orbital edema, intra-ocular lens, etc.)
Transcranial Doppler ultrasound	Primarily vascular diseases	Can pick up early signs of vasospasm, limiting the need for invasive testing	Operator dependent, limited by bone structures for acoustic windows, with limited utility for distal vessels
Near-InfraRed spectroscopy	All neurological disorders	Simple, continuous monitoring is possible, limiting the need for blood transfusions	Measurements are only superficial, limited by scalp injury and cranial defect
qEEG	Primarily epileptic disorders but also useful in vascular diseases	Allows for continuous monitoring, is relatively objective as compared to traditional EEG, and requires limited training	A propensity for false interpretation. A review by a neurophysiologist or epileptologist is still needed
Brain tissue oxygenation monitoring	Primarily TBI	Targeted monitoring of intracerebral oxygenation, allows for continuous monitoring	Invasive oxygenation measurement is limited to the focal area (15 mm^2^) around the probe
Intracranial pressure probes	All neurological disorders	Direct monitoring of ICP and CPP. EVDs can also be therapeutic. Allows continuous monitoring	Invasive. Dependent on the availability of surgeons for placement. Also limited by lack of recalibration and zero drift
Cerebral blood flow (CBF) monitoring	TBI, SAH	Monitor blood flow to the cerebral cortex, allowing for continuous monitoring. Can be recalibrated	Invasive. Also prone to drift. Limited data available
Cerebral metabolism monitoring	TBI, SAH	Direct measurement of neurotoxic metabolites in the brain	Invasive. Limited data available
Combined invasive probes	TBI	Multimodal analysis of ICP, CPP, and PBO2.	Invasive, cost-prohibitive, and limited data available

##### 2.1.6.2. Don'ts

Providers should not make any changes to the plan before comparing the examination with previously documented tests. Pretest probability and the appropriateness of testing ensure judicious use of resources. Scattershot testing can be costly and cumbersome and lead to false positives and unnecessary interventions. Hourly neurological assessment may be useful in the early phases of neurological injury to detect signs of neurological deterioration but should not be continued for prolonged periods as it interferes with sleep hygiene and may lead to delirium in these patients (12–14) (Table 2).

### 2.2. Pupillometry

The assessment of pupillary response is vital in the neurological examination in critical care (discussed in detail in Section 2.1.4 above). Traditionally, this has been done with a flashlight at the bedside, and the interpretation of size and reactivity has been left to the examiner's judgment. Modern automated pupillometers (APMs) provide accurate and reliable evaluation of the pupillary response, eliminating inter-examiner variability. The pupil response has four dynamic phases: response latency, maximum constriction, pupil escape, and recovery. These parameters can be measured quantitatively using modern pupillometers, whereas the traditional assessment is more qualitative. Automated pupillometry provides quantitative values for latency, constriction velocity (CV), and dilation velocity (15). Latency is a measure of the time from stimulus to the onset of pupillary constriction and is measured in milliseconds. Constriction velocity and dilation velocity are measures of the constriction and recovery phases and are typically reported in millimeters per second. Constriction is maximal during the initial constriction. Maximum constriction is followed by pupil escape, where the pupil reaches a state of partial constriction that persists until the light stimulus is removed. The modern hand-held pupillometers are devices with a built-in high-speed infrared camera, LED light source, and automated computation software that measure the change in pupil size at short intervals to produce quantitative results that limit individual variation during assessment (15). Neuroptics pupillometers also report the Neurological Pupil Index (NPi), a proprietary formula for assessing pupil function that has been widely used in various clinical studies (15). NPi >3 is reported as normal; NPi <3 is reported as sluggish, and NPi of zero is reported as absent. However, variations in NPi based on iris color have been reported (16). Nevertheless, the pupillary light reflex, when assessed using the pupillometer, is not dependent on iris color (16). NPi has also been reported to be affected by ambient light levels (17).

#### 2.2.1. Dos

The reproducibility and accuracy of APM have been widely studied in various clinical scenarios, including TBI, ischemic and hemorrhagic stroke, seizure, cardiac arrest, liver transplantation, brain death, drug effects, etc. (15, 18–20) and have been found to be reliable tools in critical care. The use of APMs can significantly reduce inter-examiner variability (21). While traditional examination does not reliably detect amplitude changes smaller than 0.3 mm, APM does and therefore may detect changes in clinical status earlier (22, 23). The effects of medication should always be considered when interpreting changes in pupillometric analysis. Efforts should be made to standardize ambient light conditions during pupillometric examinations to minimize the effect on NPi (17) (Table 2).

#### 2.2.2. Don'ts

There are limitations to the use of APM. It does not take into consideration changes in pupil shape, such as oval or tonic pupils, which are clinically relevant and can be detected by traditional examination. Also, the examination of agitated patients can be extremely difficult using a pupillometer (21). Periorbital and scleral edema, intraocular lenses, and changes in ambient light can interfere with pupillometric analysis (23). The pupillary response also varies with age, refractive error, and neurodegenerative diseases (24, 25). The effects of these factors have not been studied in the interpretation of automated pupillometric analysis in critical care. An NPi of zero indicates a non-reactive reactive pupil. This, however, does not take into consideration pupil size and shape. A non-reactive pinpoint pupil has a different interpretation than a mid-dilated and dilated fixed non-reactive pupil or an irregular pupil with or without segmental contraction. The interpretation of the pupillary reaction is more complex than simply measuring the NPi. Pupillometry should therefore be used as a complement to conventional examination to minimize inter-rater variability and to detect small and early changes in pupillary response, but not as a substitute for expert clinical judgment (Table 2).

### 2.3. Transcranial Doppler ultrasound

Transcranial Doppler (TCD) ultrasound is an inexpensive, non-invasive, non-ionizing, portable, and replicable bedside diagnostic tool that provides an evaluation of cerebral hemodynamics in real-time. Since its introduction into clinical practice in 1982, TCD has become a staple diagnostic tool in the field of neurocritical care (26). TCD evaluates cerebral blood flow parameters in the basal arteries by using low-frequency ultrasound waves. These parameters, together with information obtained from other imaging modalities, allow the intensivist to monitor changes occurring in the cerebral vasculature secondary to various cerebrovascular disorders or responses to the provided interventions. The basic principle of insonating basal arteries to obtain cerebral blood flow parameters is based on the Doppler effect (27). The ultrasound waves emitted by the probe are reflected by the moving erythrocytes in the blood vessels (28). The difference between the emitted and reflected frequencies is called the Doppler shift. The greater the Doppler shift, the higher the blood flow velocity in the insonated blood vessel. Because blood flow in the blood vessels is laminar, the recorded Doppler signal forming the spectral waveform on the TCD monitor is a summation of different Doppler frequency shifts reflected from different erythrocytes (29). Cerebral blood flow velocity (CBFV) along with other cerebral blood flow parameters are then measured in the insonated blood vessels by spectral analysis. Typical cerebral blood flow parameters measured include mean maximum velocity (V mean), peak systolic velocity (PSV), end-diastolic velocity (EDV), pulsatility index (PI), and resistance index (RI) (30). The TCD examination is performed with low-frequency (2 MHz) ultrasound probes because higher-frequency probes, typically used for extracranial Doppler studies, are not able to penetrate the skull. Moreover, even with lower frequency probes, TCD can only be performed at specific anatomical landmarks where the thinner skull or naturally occurring foramina allow for better ultrasound wave penetration. These areas are called acoustic windows. There are four acoustic windows used to insonate basal arteritis: transtemporal, transorbital, suboccipital, and submandibular. There are two types of transcranial Doppler devices used to identify which arteries are being insonated in specific acoustic windows: non-duplex (non-imaging) and duplex (imaging) TCDs (29). When using non-duplex TCDs, arteries are identified based on different factors, such as the acoustic window used, depth of insonation, transducer orientation, the direction of blood flow, and measured mean flow velocities. In contrast, transcranial color-coded duplex (TCCD) imaging combines the aforementioned factors with a cross-sectional view of the insonated area, which visualizes different anatomic structures and aids in the identification of specific blood vessels.

#### 2.3.1. Dos

TCD has been shown to be a valid and reliable diagnostic tool in a variety of cerebrovascular disorders, including acute ischemic stroke, subarachnoid hemorrhage complicated by cerebral vasospasm, traumatic brain injury, sickle cell disease, cerebral venous sinus thrombosis, intracranial atherosclerosis, and cerebral microemboli, and may be used as a supplementary test in the investigation of brain death. However, one of the most common indications for the use of TCD monitoring is the evaluation of vasospasm in patients with aneurysmal subarachnoid hemorrhage (SAH). Symptomatic vasospasm develops in 20–40% of patients with aneurysmal SAH and remains the leading cause of morbidity and mortality in this patient population (31). Diagnostic cerebral angiography remains the gold standard for detecting vasospasm, but it is an invasive procedure, which limits its usefulness for dynamic monitoring. TCD, on the other hand, allows daily monitoring of vasospasm and assessment of treatment efficacy. TCD has been shown to have high specificity and sensitivity for detecting middle cerebral artery (MCA) and basilar artery (BA) vasospasm when compared to conventional angiography (32), although sensitivity and specificity decrease with distal vasospasm. When there is a decrease in the diameter of a blood vessel, there is an associated increase in velocity. This is based on Bernoulli's principle, where the same amount of blood tries to pass through narrower arteries, leading to an increase in velocity and a decrease in pressure. The ability to detect these focal increases in velocity is the main utility of transcranial Doppler examinations (33). It has been determined that flow velocities >120 cm/s are indicative of mild (<25%) and those higher than 200 cm/s are indicative of severe vasospasm (>50%) of the MCA (34). Similarly, flow velocities >85 cm/s in the basilar artery have been reported to indicate moderate to severe (25–50%, >50%) vasospasm (35). However, elevations in mean blood flow velocities can be caused by multiple pathologic processes other than vasospasm, like focal stenosis, or hyperemia. One of the ways to differentiate vasospasm from hyperemia is to calculate the Lindegaard Ratio (LR), which is based on the principle that vasospasm causes a segmental or diffuse increase in flow velocities in the affected vessel without a parallel increase in flow velocities of feeding extracranial arteries such as the internal carotid artery (ICA) or vertebral arteries (VA) (27). The Lindegaard Ratio is defined as the ratio of the mean velocities of the MCA to the ICA. A ratio <3 is consistent with hyperemia, as the latter would cause a similar increase in MCA and ICA flow velocities, whereas a ratio >3 is more consistent with vasospasm (36). LR between 3 and 6 has been associated with mild vasospasm and LR > 6 is indicative of severe vasospasm. Like the Lindegaard ratio, the ratio of basilar artery mean flow velocity (MFV) to vertebral artery MFV >2, in conjunction with elevated mean flow velocities >85 cm/s, has been used to determine the presence of basilar spasm (30). However, TCDs have failed to maintain similarly consistent measurements when assessing the remaining basal arteries and several studies have revealed lower sensitivities and specificities when evaluating the anterior cerebral arteries (ACAs) or posterior cerebral arteries (PCAs) (30) (Table 2).

TCD can also be used to predict elevations in ICP by showcasing characteristic patterns of alterations in cerebral blood flow and increases in pulsatility index (PI). Elevated ICP initially causes an increase in PSV due to external compression of the arteries that narrows their diameter, while it leads to a progressive decrease in EDV and eventual reversal of flow due to unopposed external pressure from elevated ICP during diastole (37). Since TCD has a limited ability to directly visualize the distal vasculature, the pulsatility index (PI) has been used as a surrogate marker to provide information about downstream vascular resistance. It is equal to (PSV-EDV)/MFV and normally ranges from 0.5 to 1.19 (38). Proximal vessel occlusion or vasospasm typically leads to a decrease in the pulsatility index due to downstream arteriolar dilatation, whereas distal vessel occlusion/vasospasm or compression from elevated ICP, typically increases PI (36). There have been studies that have demonstrated a statistically significant linear association between PI and ICP between 15 and 40 mmHg with high sensitivity and specificity (39). Bellner et al. developed a formula (ICP = (10.93 × PI) – 1.28), which converts PI to ICP with a sensitivity and specificity of 89 and 92%, respectively (40). This TCD parameter can be used as an adjunct in the evaluation of elevated ICP, but it cannot replace direct ICP monitoring modalities in patients with traumatic brain injuries or aneurysmal SAH. TCD monitoring also has a promising role in the diagnosis and management of patients with acute ischemic stroke (AIS). It has been demonstrated that TCD can accurately identify acute occlusion in MCA with high sensitivity/specificity, positive predictive value (PPV), and negative predictive value (NPV) >90%, while for ICA siphon, VA, and BA occlusions, specificity/NPV remain equally high, but sensitivity/PPV have been reported to be between 70 and 90% (41). Moreover, TCD is also useful for prognostication, as studies have shown that normal cerebral blood flow parameters 6 h post-stroke are associated with early functional improvement (42). TCD can also be used to identify potential etiologies. For example, in patients with extra- or intracranial atherosclerotic disease, detection of microemboli on the ipsilateral side may be diagnostic of the symptomatic large-vessel disease. In contrast, the presence of microemboli in multiple arterial territories would suggest a cardiogenic rather than an atheroembolic source (43). As microemboli travel through the insonated blood vessels, they cause brief disruptions in the ultrasound waves, resulting in high-intensity transient signals (HITS) on the TCD spectral waveform (27). A similar phenomenon allows the detection of paradoxical emboli when evaluating for the presence of a patent foramen ovale (PFO) in patients presenting with a cryptogenic stroke (44). Hereditary hemorrhagic telangiectasia (HHT) has been commonly associated with PAVF, so TCD may also be a useful tool for screening this patient population (45). As intracranial vessels are being insonated, patients are injected with agitated saline to identify microemboli presenting as HITS. If present, this suggests right-to-left shunting, from either extra- or intracardiac pathologies. Unlike transesophageal echocardiography (TEE), which remains the gold standard for detecting PFOs, TCD monitoring allows patients to remain awake and participate in the performance of the Valsalva maneuver. This allows TCD to better detect PFOs that require extreme right-to-left atrial pressure gradients to open (46). TCD monitoring also has a prominent role in the management of people with sickle cell disease (SCD). In people with SCD, abnormal erythrocytes tend to adhere to the endothelium, which leads to stenosis or occlusion. It has been shown that MFV >200 cm/s in either the ICA or MCA is associated with an increased risk of ischemic stroke in children with SCD. Treatment with prophylactic transfusions results in a significant reduction of stroke risk by more than 90% (47). This has led to the inclusion of TCD monitoring in the routine screening of this patient population. In addition, TCD has recently been used to guide the safe transition of patients from chronic infusion therapy to hydroxyurea for primary stroke prevention (43).

#### 2.3.2. Don'ts

Transcranial Doppler is a valid and effective diagnostic tool that provides complementary information to other diagnostic modalities available for multimodal monitoring of a variety of cerebrovascular disorders, but it also has some limitations. First, TCD allows only limited direct anatomic visualization of cerebral blood vessels. Identification of the artery of interest is based on a variety of surrogate factors, as explained earlier. This makes the TCD examination highly operator-dependent. One of the essential technical factors is to always keep the angle of insonation relative to the direction of the vessel at <30 degrees, as this minimizes errors in flow velocity calculations. Lack of good acoustic windows can also be a challenge, especially for elderly women. Almost 10% of the general population does not have acoustic windows (48). The clinician should also be aware that in addition to underlying pathological processes, many physiological factors can also affect hemodynamic parameters such as flow velocity. These include, but are not limited to, age, gender, arousal state, CO2 or O2 partial pressure variation, blood pressure changes, hematocrit, blood viscosity, and pregnancy (30). Moreover, sensitivity and specificity for detecting vasospasm in the cerebral vasculature are not evenly distributed among the basal arteries, with the MCA being more reliable. The overall sensitivity of TCD for identifying patients at risk for delayed cerebral ischemia (DCI) after SAH, has also been shown to be poor (49). Hemodynamic measurements in ACA or PCA were less sensitive and specific for vasospasm. This makes the exclusion of vasospasm challenging, as it is essential to insonate all vessels to reliably exclude vasospasm (Table 2).

### 2.4. Near-infrared spectroscopy

Near-infrared spectroscopy is a non-invasive method for monitoring regional cerebral oxygenation (rSO2). It provides an indirect assessment of cerebral perfusion. It has been found to correlate with other techniques, viz., CT brain perfusion, brain tissue oxygen tension, jugular venous oxygen saturation and carotid doppler (50–52). It analyzes local tissue oxygenation by measuring the ratio of the concentration of oxyhemoglobin (O2Hb) to the sum of the concentrations of reduced hemoglobin (Hb) and O2Hb, similar to a pulse oximeter (53). It does this by measuring the dispersion and absorption of monochromatic light of a specific wavelength. The absorbance of light is directly proportional to the concentration of a given substance in the solution and the length of time the solution has been passed through. From the differential absorbance of different wavelengths by Hb and O2Hb, the ratio between the two in a tissue can be estimated (53). Traditional oximetry is based on measuring the change in light intensity as it passes through the entire thickness of the tissue. However, the volume of tissue in transcranial measurements makes this impractical. NIRS oximeters use adhesive patches with light sources of predetermined near-infrared wavelengths and light detectors located at fixed distances from the light source that measure the variable dispersion or scattering of the light as it traverses the tissue over short lengths of nearby tissue.

#### 2.4.1. Dos

rSO2 is measured by placing NIRS patches bilaterally on the forehead. Comparisons of changes in rSO2 are more clinically relevant than absolute values. A reduction of more than 20% in rSO2 from baseline is associated with worse outcomes (54, 55). NIRS oximetry can be used as a tool to predict outcomes in critically ill, comatose patients (56). Targeting transfusion goals at a combination of hemoglobin level and rSO2 goal of 60% may help reduce blood transfusion rates in critically ill patients in the neuro-ICU (57). NIRS oximetry may be useful during cardiac arrest and in post-arrest care (58, 59). NIRS can be concurrently used with EEG, as its near-infrared light does not interfere with the electrical EEG measurement, and hybrid systems are being developed (60) (Table 2).

#### 2.4.2. Don'ts

NIRS patches only measure about 2 cm below the light source, so it should not be assumed that it will measure all areas of the brain. The most common place to place NIRS patches is on the hairless frontal forehead region, which is a “watershed” region of the brain supplied by the anterior cerebral artery (ACA) and middle cerebral artery territories, and these areas are supplied by the ipsilateral carotid artery. Therefore, clinicians observing ACA-MCA vasospasm after subarachnoid hemorrhage can monitor that since TCD sensitivity is not the best for ACA vasospasm compared to MCA-M1 segments. However, frontal NIRS patches will not give meaningful information on most patients with basilar vasospasm or similar vascular territory. In patients with scalp injury and skull discontinuity, NIRS may not be a reliable tool as these conditions can potentially affect the dispersion of the infrared signal, interfering with the determination of rSO2 measurements (61) (Table 2).

### 2.5. Quantitative EEG

Seizures and status epilepticus, especially non-convulsive seizures/status, are common in critically ill patients with an incidence of 8–40% (62). Uncontrolled seizures are associated with physiologic and structural abnormalities (63). Irregular movements in the ICU are common and often have a non-epileptogenic etiology. Nonetheless, such non-epileptic movements have significant overlap with various non-convulsive seizure semiologies (64). As such, continuous EEG monitoring (and, by extension, quantitative EEG) is an important tool in the evaluation of patients in the neuro-ICU. Indications for EEG monitoring in the ICU include detection of non-convulsive seizures, characterization of seizures, monitoring of ongoing therapy (i.e., induced coma or level of sedation), detection of ischemia, and prognosis (i.e., cardiac arrest or brain injury) (65). Quantitative EEG (qEEG) could be useful in all of the above situations because it can provide more effective real-time monitoring.

qEEG is often presented as a series of “panels”. These panels are often shown as two-dimensional representations of three-dimensional graphs with the *x*-axis as time, the *y*-axis as a measurement/calculation (i.e., frequency, amplitude, ratio, or percentage), and the *z*-axis as another measurement displayed as a color scale. Commonly used panels show the probability of seizure (automated seizure detection by a proprietary calculation), areas of high amplitude and rhythmicity at a given point in the EEG (left/right rhythmicity spectrogram), the amplitude of various frequencies (left/right spectrogram), asymmetry in frequency, asymmetry in amplitude (asymmetry indices), the minimum and maximum amplitudes for each epoch (amplitude integrated EEG), the degree of burst suppression (suppression index), and a magnified view of the difference between slow and fast frequencies (alpha-delta ratio). The qEEG panel display shows multiple calculations over several hours of raw EEG data. As with any monitoring tool (and the raw EEG data from which it is derived), there are clinically relevant patterns, misleading artifacts, and pitfalls that require attention and experience to decipher. Newer mathematical models, artificial neural networks, and machine learning methods are now being used to create algorithms for seizure detection, artifact detection, and more. Seizure detection, burst suppression, and ischemia detection on a modern qEEG display can be identified by non-epileptologists and/or non-neurologists with minimal training. This could at least prompt a page to the on-call EEG reader for further review if not immediate treatment in the proper context. Otherwise, the EEG continues to run, capturing important abnormalities that may not be noticed until the next morning, when the epileptologist starts reading the overnight backlog of EEGs.

Compared to conventional neurophysiologist review, qEEG is sensitive to seizures (87.3%), periodic epileptiform discharges (100%), rhythmic delta activity (97.1%), focal slowing (98.7%), generalized slowing (100%), and epileptiform discharges (88.5%) (66). In at least one study, no difference was found between neurologists, neuro-ICU nurses, and EEG technologists in interpreting qEEG data (67). Conversely, seizures with certain characteristics make them more likely to be missed on qEEG (i.e., <1–2 min, slower evolution, <75 μV amplitude) (68). Also, rhythmic or periodic EEG patterns that are non-ictal are often a source of false positives with qEEG, or the evolution of a rhythmic pattern into a seizure is missed by qEEG seizure detection.

As cerebral blood flow decreases, EEG changes are evident within seconds before clinical manifestations of ischemia begin. Alpha and beta frequencies decrease, followed by increases in delta and theta frequencies while the ischemia may still be reversible. As the ischemia becomes irreversible, all EEG frequencies are attenuated. Depending on the qEEG system being used, these data may be represented as an asymmetry index, a relative alpha variability, or an alpha:delta ratio (69). Subtle changes in the relative delta/theta vs. alpha/beta frequencies are more readily apparent in qEEG panels, especially if they change slowly over time. This is most commonly used to detect delayed cerebral ischemia after an aneurysmal subarachnoid hemorrhage but may be useful in other clinical scenarios. Unlike commonly used vascular imaging modalities such as TCD, CT angiogram with perfusion, or digital subtraction angiography, qEEG provides real-time monitoring rather than snapshots in time.

Although not currently in widespread use, invasive EEG monitoring (i.e., depth electrodes, subdural electrodes, etc.) allows the detection of abnormalities not detected by surface electrodes or before they are evident on surface electrodes. In critically ill patients with brain injury, invasive monitoring allows the detection of depth-only seizures, earlier detection of seizures as compared to surface electrodes, and early detection of evolving ischemia (70). In addition, subdural electrodes are more likely to detect spreading depolarization and can be more readily detected in brain injury (71), which has been associated with worse outcomes in several pathologies in the neurocritically ill (72).

#### 2.5.1. Dos

Use continuous EEG for non-convulsive seizure detection, seizure characterization, monitoring of induced coma therapy /level of sedation, ischemia detection, and prognostication when indicated. For quick screening of the EEG record, qEEG can be used, but a review of the raw EEG is always recommended later. qEEG may be used for minute-to-minute bedside monitoring in situations where continuous EEG is indicated. These data can be interpreted by non-neurologists, provided they have some level of training and backup. qEEG may be used for ischemia detection in aneurysmal subarachnoid hemorrhage by looking at alpha: delta ratios, asymmetry indexes, or relative alpha variability, depending on the EEG software used. Ischemia detection with qEEG may be considered in other situations where there is a high risk of cerebral ischemia and early detection of ischemia could aid in clinical management or prognosis (i.e., cerebrovascular surgery, carotid intervention, patients at high risk of recurrent stroke in the short term) (Table 2).

#### 2.5.2. Don'ts

qEEG should not be used as the sole method of EEG interpretation, and its automated seizure detection should not be over-relied upon. The raw EEG data should always be reviewed or examined by a neurophysiologist before deciding that an event had a true electrographic correlation. It should be recognized that rhythmic or periodic EEG patterns are either misinterpreted as seizures or mask the evolution of seizures in qEEG automated seizure detection. In this context, the qEEG panel should not be blindly trusted and continuous EEG should not be left on without reason. If a seizure has been detected and characterized, it should be considered whether the patient still needs continuous EEG. At the same time, qEEG and automated detection should not be disregarded, as the technology has gotten better over time and will continue to do so. qEEG is often used as a roadmap to “see the forest for the trees”, with raw EEG being the trees. Ischemia detection footprints on raw EEG are easily overlooked, even by experienced neurophysiologists. The raw EEG read for ischemia detection should not be relied upon. qEEG data correlated to the clinical situation by the bedside neurointensivist can make a big difference, especially in low-grade aneurysmal subarachnoid hemorrhage (Table 2).

### 2.6. Brain tissue oxygenation (PbO2) monitoring

Maintaining adequate oxygenation of the brain is the cornerstone of most of the interventions provided in neurocritical care, so it would be logical to use brain tissue oxygenation along with other forms of multimodal monitoring as part of the outcome measure. However, it was not until 2007 that brain tissue oxygenation (PbO2) monitoring was included in the guidelines for the management of patients with severe traumatic brain injury (73). There are two commercially available probes for invasive brain oxygenation monitoring, Licox, and Neurovent-PTO, although Licox is more commonly encountered in clinical practice. Both probes are designed to measure oxygen saturation in the surrounding white matter, however, they cannot be used interchangeably as significant differences in PbO2 have been reported when comparing the two devices (74). The technology behind the indirect measurement of tissue PbO2 is based on diffusion. Oxygen diffuses through a permeable membrane into the electrolyte solution-filled catheter tip. The higher the tissue oxygen partial pressure, the more oxygen gets diffused into the probe, which leads to the formation of an electrical current proportional to the oxygen partial pressure (75). This process is affected by the temperature, so a temperature probe is typically also included in the device. The normal range for tissue oxygenation is approximately 23 ± 7 mm Hg (76). Values below 20 mmg Hg have been associated with poor outcomes in patients with traumatic brain injury and have been therefore used as a marker for therapeutic intervention, while values below 10 mm Hg are already considered a critical level of cerebral ischemia (77). Oxygen delivery to the tissues depends on the perfusion of that organ and the oxygen content of the blood. In the case of the brain, this includes cerebral blood flow (CBF), which is affected by cerebral perfusion pressure (CPP). This is why brain tissue oxygenation is significantly affected by CPP, and in clinical practice, it can be used as a marker to determine the lowest acceptable limit of CPP in patients with traumatic brain injury. However, the second part of this equation also includes the oxygen content of the blood and the tissue delivery process, which, if impaired, can negatively affect PbO2 even in the presence of normal CBF/CCP. Therefore, interventions to improve PbO2 include not only increasing CPP, but also adjustments to improve the partial pressure of oxygen (PaO2) or increasing hemoglobin by blood transfusion (78). Newer emerging methods of monitoring brain tissue oxygenation, such as Near Infrared Spectroscopy (NIRS) can be used for non-invasive measurements (as described above). The normal range is reported to be ~60–75%, but due to variability in baseline levels it is more useful for monitoring the overall trend.

#### 2.6.1. Dos

The pathophysiology of brain injury in patients with severe traumatic brain injury (TBI) has been described to be 2-fold, first from a primary traumatic insult followed by secondary brain injury (SBI) from subsequent inflammation with increased vascular permeability, mass effect swelling, tissue ischemia, and oxidative stress (74). There have been multiple studies performed over the years to assess the role of PbO2 monitoring and its effect on outcomes in patients with severe TBI, although the results have been variable (73, 76, 78–80). Overall, the majority of studies suggest a favorable effect of combined ICP + PbO2 monitoring therapy on functional outcomes in patients with severe TBI. The placement of the PbO2 monitor has been reported to be easy and safe, with a very low complication rate. It needs to be calibrated at room temperature before insertion, and a waiting period of ~1 h is recommended before utilizing the measurements for decision-making. The PbO2 monitoring catheter is placed in the brain parenchyma at ~2.5–3 cm depth from the dura, typically in normal-appearing brain tissue on the side of the injury. In diffuse cerebral injury, the non-dominant hemisphere is preferred for catheter placement (81). Regarding NIRS, its use in neurocritical care for the management of patients with severe TBI has been limited, possibly due to surface signal distortion from underlying cutaneous, epidural, or subdural fluid collections/hematomas. However, it remains a promising diagnostic modality due to its ease of use, non-invasive nature, and minimal inter-operator variability (82) (Table 2).

#### 2.6.2. Don'ts

Invasive PbO2 monitoring has its disadvantages. Given its invasive nature, there is a risk of hemorrhage and infection, although this has been reported to be < 1% (77). Moreover, due to the placement of the probe in the parenchyma, typically at the side of the lesion, the measurements provided may not reflect a global picture of brain tissue oxygenation. The device typically measures approximately an area of 15 mm^2^ of white matter around the catheter tip, thus measuring the focal PbO2 of the area at risk, unless there is diffuse injury (75). Some of these drawbacks may be associated with overtreatment, which can lead to hyperoxia and subsequent complications including impaired oxygen autoregulation due to cerebral vasoconstriction or acute lung injury (81) (Table 2).

### 2.7. Intracranial pressure only probes

Intracranial pressure (ICP) monitoring is one of the most common forms of multimodal monitoring currently utilized in neurocritical care today, and is included in the Brain Trauma Foundation's guidelines for the management of severe traumatic brain injury. The concept behind ICP monitoring is based on the widely accepted Monro-Kellie doctrine. The latter states that ICP is generated by positive pressures from brain parenchyma, cerebrospinal fluid, and blood volume. These compartments are located within a non-compressible cranial defect, so an increase in one should be compensated for by a decrease in another to maintain ICP within the accepted range. There is a volume reserve in the intracranial space, which is ~60–80 ml in adults (83). Due to progressive atrophy of the brain, the reserve volume increases with age. However, when this compensatory mechanism or reserve volume is exhausted, intracranial pressure increases as a result. Severely elevated intracranial pressure represents a neurologic emergency, as it can lead to a significant mass effect with subsequent herniation, or cause secondary brain injury due to cerebral ischemia from decreased cerebral perfusion pressure (CPP), ultimately leading to death. Such catastrophic increases in ICP can occur in patients with severe traumatic brain injury, space-occupying lesions, large ischemic strokes, intraparenchymal hemorrhage, or subarachnoid hemorrhage, which would explain the role of continuous ICP monitoring in these patient populations. There are different types of invasive ICP monitoring devices available. The most commonly encountered device is the external ventricular drain (EVD) system, which is placed intraventricularly and monitors global intracranial pressure. One of the advantages of EVD placement is that it allows for therapeutic intervention while providing continuous ICP monitoring. However, there are a few clinical scenarios where EVD placement is not plausible, such as in cases of obstructive/non-communicating hydrocephalus, slit-like ventricles, severe brain swelling, or midline shift, which make placement challenging. Such cases call for alternative invasive ICP monitoring devices, including intraparenchymal, epidural, subdural, or subarachnoid space probes, commonly referred to as “bolts”. These monitoring modalities provide isolated continuous ICP monitoring without therapeutic capabilities. Currently, there are a few different types of micro transducer devices available on the market and they are based on three main pressure monitoring technologies, including fiber optic, piezoelectric strain gauge, and pneumatic sensors (84).

The pressure reactivity index (Prx) is a tool used to measure an individual's state of cerebral autoregulation. It is the rolling correlation between ICP and mean arterial blood pressure (MAP)/Cerebral perfusion pressure (CPP) and is typically measured sequentially over periods of 10 s (85). This allows for filtering out factors responsible for more rapid changes in the waveforms over a short period of time (i.e., respiratory and arterial pulsatility-related variations), but can be evaluated over longer periods and is referred to as L-Prx. Positive Prx values indicate impaired cerebral autoregulation, while negative values indicate intact cerebrovascular reactivity. Prx/L-Prx is a strong indicator of outcome in neurological injuries such as TBI, SAH, etc. (86, 87). It can also be correlated with the non-invasive transcranial Doppler mean flow index (88) and the near-infrared spectroscopy hemoglobin volume index (HVx) (89). PRx correlates with changes in ICP and suggests a worsening of autoregulation with increasing ICP, potentially allowing the establishment of an individualized threshold for harmful ICP for patients in neurocritical care units (90).

#### 2.7.1. Dos

The Brain Trauma Foundation has included continuous ICP monitoring in its guidelines for the management of patients with severe traumatic brain injury, specifically those with a Glasgow Coma Scale (GCS) of 3–8, and an abnormal computed tomography (CT) scan (73). Abnormal CT scan findings include hematoma, contusion, swelling, herniation, or compressed basal cisterns. ICP monitoring has also been recommended in patients with traumatic injury and a normal CT scan if two of the following criteria were met on admission: age >40 years, unilateral or bilateral motor posturing, or systolic blood pressure (BP) <90 mm Hg (73). These recommendations are often adapted by clinicians and ICP monitoring is used in a variety of clinical scenarios (91). Among the available ICP-only monitoring devices, intraparenchymal probes are the most commonly used. The most reliable device for ICP monitoring remains the EVD, but there have been several studies that have shown that the accuracy of intraparenchymal pressure monitoring devices is also reliable and provides equivalent measurements when compared to intraventricular monitoring devices (92). Intraparenchymal devices are easier to place and have a lower risk of developing hemorrhage or infection. They are also less likely to become occluded by bleeding or debris. The location for placement of the intraparenchymal catheter is variable, preferably at the site of the lesion to avoid overestimation of CPP, with the tip typically advanced up to 5 cm into the brain parenchyma (75). These types of devices require only one-time calibration and zeroing prior to placement and, unlike EVDs, intracranial pressure readings are not dependent on patient position (93). In addition, because they are not fluid-coupled devices, the incidence of pressure waveform attenuation and measurement artifacts is lower (83). Similar advantages apply to other ICP-only monitoring devices, such as epidural, subdural, or subarachnoid bolts, although their pressure readings have been reported to be less reliable and they are more susceptible to blockade by debris (Table 2).

#### 2.7.2. Don'ts

As discussed above, invasive ICP-only monitoring devices have their advantages and disadvantages. The epidural, subdural, or subarachnoid bolt devices have been shown to be less accurate and therefore rarely used. While intraparenchymal devices provide reliable ICP readings, the lack of recalibration capabilities makes them more prone to zero drift with prolonged use. The Spiegelberg transducer, which uses a pneumatic sensor for ICP measurement, has incorporated auto-calibration capabilities to try to address this disadvantage seen in other micro transducers (84). Multiple studies have evaluated the accuracy of ICP-only probes over the years, with few showing a positive correlation between the degree of drift and the duration of use (94, 95). The duration of monitoring, however, has been variable, with some papers reporting zero drift as early as the first 24 h (96). The most recent meta-analysis by Zachetti et al. regarding the accuracy of ICP monitoring revealed that the mean drift for ICP-only probes was <1 mm Hg over the long period of observation and that there was no statistically significant correlation with the duration of monitoring, contrary to what was previously believed (97). Furthermore, there is a possibility that pressure readings from intraparenchymal devices may not reflect global ICP, but rather the interhemispheric ICP gradient commonly seen in focal intraparenchymal lesions (75). The lack of CSF drainage capabilities and high cost also limit the use of these types of ICP-only monitoring devices in the neurocritical care unit. The use of Prx to calculate optimal CPP requires a sufficiently large span of CPP change, which may not be captured using fixed, relatively short periods, limiting its use in emergent situations (Table 2).

### 2.8. Cerebral blood flow monitoring

The brain depends on a constant supply of oxygen and glucose through cerebral blood flow to maintain metabolic function and structural integrity. Measurements of CBF are relevant in conditions where alterations in flow dynamics and autoregulation may lead to cerebral ischemia and infarction. The information obtained from the measurement of CBF is different from that of PbO2 because it does not monitor oxygen content, but it can help clarify when tissue hypoxia is related to oligemia or hyperemia. In addition, measurement of CBF have been used to measure the effects of cerebral vasospasm. Historically, the methods used to measure CBF were “snapshots in time”. Single-time measurements with the use of various tracer gases, dyes, and thermodilution techniques were the predominant techniques; however, the current multimodal approach to monitoring the neurocritically ill patient requires that measurements be continuous or at the very least near-continuous, and applicable at the bedside (98). Currently, no continuous, non-invasive techniques for monitoring CBF exist. TCD, its applications for measurement, and its drawbacks as a monitoring tool have been discussed previously in this paper. At present, all bedside methods are limited to regional blood flow and do not consider global CBF. A common invasive method to assess regional blood flow uses thermal diffusion to measure local CBF, or so-called thermal diffusion flowmetry (TDF). It uses an intraparenchymal probe consisting of a thermistor and a temperature sensor. The probe measures the thermal gradient between the distal thermistor, which is heated by 2 degrees Celsius, and the proximal temperature sensor (99). By tracking changes in power, a quantification of regional CBF is quantified in mL/100 g/min. This method has been validated using xenon-enhanced CT, which is regarded as the gold standard in CBF and is typically used to validate other perfusion methods. As brain edema is an important cause of neurological deterioration, one study has provided some evidence that real-time monitoring of brain water content is feasible using thermal conductivity (100). However, further studies are needed to determine the clinical utility of this method.

#### 2.8.1. Dos

TDF may be useful in aneurysmal SAH to detect changes in regional blood flow and monitor response to therapy. In one study, the TDF method was found to have a 90% sensitivity and 75% specificity for detecting vasospasm (101). TDF may be useful after TBI to detect regional changes in CBF and monitor response to therapy. TDF may be useful in assessing cerebral autoregulation. TDF can also be used to assess cerebrovascular reactivity to PaCo2, which may be useful for targeting hyperventilation in patients with altered cerebral autoregulation (102) (Table 2).

#### 2.8.2. Don'ts

TDF measures only regional CBF in the immediate vicinity of the probe, so it cannot be used to make statements about global CBF. Limitations regarding the accuracy of systemic temperature fluctuations and hyperthermia have been described. Other technical limitations such as drift have also been described (103). Other considerations include the need for automatic recalibration every 30 min, which contributes to some data loss (Table 2).

### 2.9. Cerebral metabolism monitoring

The physiological proposal for obtaining metabolic data is based on the assertion that the transition point from aerobic to anaerobic metabolism represents a bioenergetic failure and may allow for further measurement, characterization, and ultimately intervention in the acute brain injury state. Cerebral microdialysis (CMD) estimates brain metabolism by measuring metabolic intermediates such as glucose, lactate, and pyruvate and other small molecules such as glycerol and glutamate (104). Glycerol can be used as a marker of cell membrane damage and glutamate can provide some insight into post-injury excitotoxicity (105). These analytes are sampled from a small amount of extracellular fluid. The microdialysis catheter is a flexible catheter with a 10 mm semi-permeable membrane that is inserted into the cerebral white matter via an intracranial bolt. The catheter is infused with a dialysate similar in composition to that of CSF, thus allowing for equilibration across the semipermeable membrane and sampling to take place.

#### 2.9.1. Dos

CMD has been mostly studied in the context of SAH and TBI. Elevated lactate and lactate/pyruvate ratios (LPR) may be markers of inadequate CBF and/or oxygen delivery. While there is no agreed-upon number for what constitutes a metabolic crisis, an LPR > 40 is often considered the threshold (106). In SAH, elevated levels of glutamate, lactate, and glucose have been associated with worse neurological outcomes (107). In one study, LPR, along with glutamate levels, was associated with increased predictability of DCI hours before symptom onset (108). In at least one study of TBI, the poor neurological outcome was associated with increased LPR and decreased glucose concentration (109) (Table 2).

#### 2.9.2. Don'ts

There is minimal evidence that the use of CMD leads to improved clinical outcomes, and guidelines suggest that if used for prognostication, it should be used within the appropriate clinical context along with other monitoring modalities (110). Regarding the underlying metabolic state, elevated lactate and LPR levels may not be related to ischemia or hypoxia but may be secondary to mitochondrial dysfunction (111). Knowledge of mitochondrial status may be a barrier to the interpretation of LPR values (112). As with other modalities, probe location matters, and the interpretation of values will depend on whether the probe is located in an area of normal-appearing tissue or in an area of dysfunction from a lesion such as a contusion (113). An important limitation of this technique is that monitoring is not continuous, and current devices require hourly dialysate analysis, which places a high burden on bedside personnel (Table 2).

### 2.10. Combined ICP and PbO2 monitoring

The combination of two or more invasive monitoring modalities is probably what most neurointensivists think of when discussing multimodal monitoring. Most of the studies completed have used multiple probes to measure ICP, PbO2, temperature, CMD, etc., but practical advances are being made. One such option for combination probes is the Raumedic Neuro-vent PTO, which is a 3-inch catheter used to measure ICP, PbOt, and brain temperature. While combination probes are certainly helpful for practicality, the most interesting concepts are what can be gained by combining modalities. A recent narrative review by Tas et al. summarized the findings of 112 multimodal studies over a 7-year period (114). The findings are indicative of several things. The most studied conditions are TBI and SAH, and currently, most studies have combined ICP monitoring with another modality, with PbO2 being the most common.

#### 2.10.1. Dos

As with the previous section on PbO2, there is mounting evidence to support the monitoring of brain tissue oxygenation. Boost-2 was a pilot RCT in TBI patients that demonstrated that protocol-driven ICP and PbO2-targeted therapy was both feasible and safe compared to ICP therapy alone (76) (Table 2).

#### 2.10.2. Don'ts

At present, the combination of modalities has not been shown to definitely improve patient outcomes. In that study, there was a non-significant trend toward the improved outcome, although importantly BOOST-2 was not powered for this outcome. BOOST-3 is currently being conducted to evaluate combined ICP and PbO2-targeted therapy in TBI (80). Another limitation of monitoring is that probe placement is not standardized. Furthermore, it is challenging to integrate data from different probes. The concurrent use of devices used for the measurement of different physiological signals is a significant barrier when each device manufacturer uses proprietary methods for collecting and converting signals or unique communication protocols (Table 2).

## 3. Future of MMM

The simultaneous use of more than one modality to predict and treat a secondary neurological injury is being investigated. Dual EEG/NIRS monitors are now commercially available and are widely used (115, 116). Triple-lumen catheters that can simultaneously collect data on intracranial pressure, brain temperature, and cerebral oxygenation have been shown to be safe (117). Data on the utility and safety of the simultaneous use of invasive and non-invasive monitoring modalities are still sparse but may yield promising results in the future. The prohibitive cost of such monitoring remains a major barrier to research and clinical use. As these technologies become more common and less expensive, research will become more feasible in the future.

## Author contributions

All authors listed have made a substantial, direct, and intellectual contribution to the work and approved it for publication.
